# Biomedical Ethical Aspects Towards the Implementation of Artificial Intelligence in Medical Education

**DOI:** 10.1007/s40670-023-01815-x

**Published:** 2023-06-07

**Authors:** Felix Busch, Lisa C. Adams, Keno K. Bressem

**Affiliations:** 1 Department of Radiology, Charité – Universitätsmedizin Berlin, Corporate Member of Freie Universität Berlin and Humboldt Universität zu Berlin, Berlin, Germany; 2 Department of Anesthesiology, Division of Operative Intensive Care Medicine, Charité – Universitätsmedizin Berlin, Corporate Member of Freie Universität Berlin and Humboldt Universität zu Berlin, Berlin, Germany; 3 Department of Radiology, Stanford University School of Medicine, Stanford, CA USA; 4 Berlin Institute of Health at Charité – Universitätsmedizin Berlin, Berlin, Germany

**Keywords:** Biomedical ethics, AI, Education, Medical, Practice guidelines, Machine learning, ChatGPT

## Abstract

The increasing use of artificial intelligence (AI) in medicine is associated with new ethical challenges and responsibilities. However, special considerations and concerns should be addressed when integrating AI applications into medical education, where healthcare, AI, and education ethics collide. This commentary explores the biomedical ethical responsibilities of medical institutions in incorporating AI applications into medical education by identifying potential concerns and limitations, with the goal of implementing applicable recommendations. The recommendations presented are intended to assist in developing institutional guidelines for the ethical use of AI for medical educators and students.

## Background

Artificial intelligence (AI) has the potential to revolutionize medicine by improving patient outcomes with personalized medicine, increasing efficiency, and reducing healthcare costs by supporting medical professionals in patient healthcare, diagnosis, decision-making, and conducting research, among many other possible applications [1–5]. Besides, there are several opportunities for how AI applications can be used to enhance medical training. Potential applications include virtual patient simulations or case studies that allow medical students to practice diagnosing and treating patients in a controlled environment, machine learning for image analysis to help interpret medical images and detect anomalies, natural language processing models for transcribing medical records or content, and tailored or intelligent learning plans and feedback based on personal strengths and weaknesses [6–11]. Moreover, medical students who have received training in AI may be better equipped and more comfortable using AI tools and technologies in their later clinical work as they learn to understand and use AI concepts and their potential applications from the beginning of their careers [12, 13]. On the other hand, large language models (LLMs) such as ChatGPT are becoming increasingly popular and are likely to revolutionize medical education in both positive and negative directions. While LLMs can explain medical terminology to students (and patients) or simulate anamnestic interviews, they could also be used to cheat on exams, papers, and assignments [14]. The increased use of AI in medicine also raises many ethical questions. Common ethical concerns about the use of AI in medicine include lack of transparency, insufficient knowledge about the application used, or false/misleading results [15, 16].

In addition, AI algorithms trained on biased data can lead to incorrect diagnoses and unreasonable or unfair decisions [17]. Hence, AI applications may perpetuate existing inequities in the medical field by providing unequal access to care or making biased treatment decisions. Further concerns include the insufficient protection of data privacy and confidentiality, as well as the lack of informed consent when retrospectively using patient data for training [18, 19]. Finally, there is a risk that AI systems will compromise patient autonomy and dignity by making treatment decisions without appropriate oversight [15, 20].

While current publications on AI-related medical education emphasize key competencies or explore educational AI programs and concepts, they fail to specifically account for the four main pillars of biomedical ethics: autonomy, justice, non-maleficence, and beneficence [21–24]. However, considering biomedical ethics when integrating AI applications into education is of particular importance, especially in the medical field, where vital measures can be taught and healthcare, education, and AI ethics collide. Therefore, this commentary emphasizes ethical issues related to the use of AI applications in the medical curriculum and proposes recommendations for medical institutions within a biomedical ethical framework.

## Biomedical Ethical Principles of Using AI in Medical Education

In general, medical institutions should ensure that they have the necessary technical infrastructure, resources, and expertise to support the use of AI in medical education. Therefore, it is required to clearly define the learning purposes and objectives when using educational AI [25]. These include general pedagogical and ethical considerations such as the pedagogical approach and the integrity of teachers and learners, as AI should enhance the classroom experience while preserving the fundamental dimensions of the human being [26]. It may be beneficial to involve all relevant stakeholders, including developers, providers, regulators, faculty, staff, medical bioethicists, and students, in the decision-making process to gather and discuss expertise, desires, concerns, ideas, and moral, legal, and ethical issues and to ensure a satisfactory implementation for all parties within possible limits [27].

This work focuses on the four main principles of biomedical ethics for medical institutions when integrating AI into medical education rather than on the general ethics of AI in education or healthcare.

### Autonomy

The principle of autonomy emphasizes the inherent and unconditional value of every human being and their right to self-determination [21, 22]. This includes the ability to make rational judgments and moral choices as well as the unrestricted right to exercise control over one’s own decisions. In medical bioethics, autonomy is a cornerstone, guiding the interactions between healthcare providers and patients and ensuring respect for individual preferences and values. However, integrating AI into medical education poses significant challenges to the autonomy of users, including students, educators, and medical professionals. For example, advanced computational models for natural language processing, such as ChatGPT, occasionally hallucinate, i.e., generate information or responses that are not based on factual data [28]. Although current technological capabilities do not allow the complete elimination of this issue, efforts can be taken to minimize its occurrence. In the context of medical education, it is crucial that LLMs possess mechanisms to transparently signal their limitations or uncertainties to prevent the propagation of erroneous information, ensuring full autonomy (and non-maleficence) among learners. The dependence on AI technology may also result in the lack of development of essential decision-making skills and clinical judgment [29]. Moreover, the complexity and opacity of AI algorithms can make it challenging to comprehend how algorithms arrive at specific decisions, potentially reducing the ability to make informed judgments about the appropriateness and correctness of AI-generated recommendations or diagnoses [30]. Therefore, medical institutions should ensure that the use of AI in the medical curriculum is transparent and comprehensible and that users know when and how different AI applications are used [31]. Furthermore, their informed consent should be obtained to ensure they fully understand and agree to use the application [32].

On the other hand, medical institutions should promote professional responsibility and accountability to empower users to make rational judgments and moral decisions, accounting for the use of AI applications with the best possible knowledge and conscience [33]. Finally, universities should offer AI models as an add-on to the medical curriculum rather than a complete replacement for traditional teaching materials and strategies so that students can decide at any time whether or not to use AI applications.

### Justice

The principle of justice comprises fairness, equity, and equal treatment for all individuals [21, 22]. This principle requires that benefits and burdens are distributed equitably among all those affected by a decision or action. Although 60% of the data used to develop AI applications is estimated to be synthetic by 2024, 40% will still be based on real data [34]. This brings forward a key question of justice — how to adequately compensate and acknowledge data sources utilized in training AI models? For medical institutions, this can be challenging to verify, especially for AI products developed by external vendors. Therefore, one potential solution might be to control data-sharing agreements between data providers and vendors before implementing AI applications. On the other hand, new laws and regulations on the use of data in AI development are needed to set standards and enforce fair practices. Furthermore, before integrating AI into medical education, an equity and social justice framework should be established to avoid a disproportionate impact on certain user populations [35]. This framework should guide the development and application of AI technologies, ensuring that they are accessible to everyone and as individualized as possible to meet the needs and perspectives of users from different backgrounds, social classes, knowledge levels, and interests to ensure that AI is used in a way that is inclusive and respectful of diversity [27, 36]. This can be reached by offering financial assistance, scholarships, or subsidies to ensure that no user is disadvantaged due to lack of access to AI applications or resources, an inclusive and adaptable design of AI applications that is developed on the needs and perspectives of a diverse set of stakeholders, or the use of diverse training data to account for equal treatment of all individuals. For example, to ensure that a broader range of perspectives is considered in the development and implementation of AI technologies, collaborations can be established with educational institutions from diverse socioeconomic backgrounds [37]. In terms of diversity of training data, institutions need to ensure that developers train their algorithms and models on high-quality, diverse data that accurately represent the population being studied so that the resulting educational content is unbiased and fair [38, 39]. This requires careful attention to data collection and curation, as well as ongoing monitoring and refinement of algorithms. Finally, AI tools that are used in medical education should undergo regular audits to detect and correct potential biases and inequities that may inadvertently arise, which also addresses non-maleficence, for example, when exploring treatment recommendations for patients [40].

### Non-maleficence

Non-maleficence is the principle that emphasizes the importance of not causing harm and minimizing potential negative consequences [21, 22]. Using AI in medicine presents both opportunities and challenges to uphold the principle of non-maleficence. Especially AI algorithms in medical education must be carefully designed, validated, and evaluated to ensure that they produce accurate and reliable results that do not mislead users and consecutively put patients at risk. Importantly, all users should be adequately educated about the application’s functionalities, known biases, and potential risks prior to using AI models, which is an essential prerequisite for all four bioethical principles [41]. For example, when AI algorithms are trained based on biased or incomplete data, they can perpetuate or reinforce existing biases and inequities in healthcare. This may lead to inaccurate or discriminatory outcomes and potential harm to specific patient groups, such as the recently discovered bias against women in AI tools [42]. To adequately assess any limitations or biases of medical AI applications, complete and detailed information and understanding of the training data for each application are required, as described above. However, this is often not applicable, for example, when applications are not open source or with missing knowledge about the technology used [43]. Therefore, medical institutions should outline the limitations of any AI application used and point out the risks of applications without transparency and of systems trained on biased or unrepresentative datasets. In this regard, medical data experts, such as physicians and medical educators, have a special ethical responsibility, as they are the ones who can detect bias in data, validate models, and train students in AI sufficiently to detect errors themselves.

On the other hand, medical institutions should provide AI-independent information to enable students to make informed decisions without the use of AI or to compare AI-generated data (which also reinforces autonomy), particularly when the results of AI applications appear to be divergent or misleading, for example, when researching diagnoses or treatment recommendations [44]. Moreover, students must be aware that AI-generated recommendations may not provide a complete and all-encompassing understanding of a patient’s unique medical situation. It also should be noted that the different AI-generated information also poses different risks when the retrieved information is applied to patients. For example, while simply retrieving information about drug interactions or dosing regimens using LLMs poses a lower risk, extended use of the models to the point of identifying symptoms, diagnosis, and treatment planning without AI-independent verification can be particularly dangerous [45].

When AI applications are used to work with real patient data, privacy and confidentially of sensitive data must be warranted, and informed consent might be required from patients whose data are being used [46]. This requires ensuring that all users adhere to strict privacy regulations and policies, for example, the General Data Protection Regulation (GDPR) and the Health Insurance Portability and Accountability Act (HIPAA) for institutions in the USA. In addition, approval from the Food and Drug Administration (FDA), European Medicines Agency (EMA), or country-specific equivalents should be obtained for each new use case, as well as approval from the internal institutional review board, including an appropriate approach to ensure privacy and confidentiality. The use of AI applications for individual medical education or knowledge acquisition will also generate new sensitive data, for instance, the assessment and analysis of individual performance, which may lead to competitive pressures or negative emotions when results are compared with those of fellow students or when faculty members are provided with access [47, 48]. Therefore, institutions should handle individual results with confidentiality. This may include decentralized storage of data and evaluation of individual skills, which can only be accessed by the respective individual.

Finally, AI in medicine is a rapidly evolving area of research. This can certainly lead to dissatisfaction and be disruptive for medical institutions. However, constantly changing applications or expanded use cases may also lead to newly identified risks or harms associated with AI, such as privacy or security concerns, discriminatory outcomes, and embedded or inserted biases [49]. Therefore, ethical guidelines should be continuously monitored and adjusted.

### Beneficence

The principle of beneficence in biomedical ethics emphasizes the obligation to promote and protect the welfare and well-being of individuals [21, 22]. In the context of AI in medical education, beneficence involves providing appropriate training on AI applications before their implementation to maximize the benefits for all stakeholders, including students, educators, and experts [7, 50]. Moreover, as outlined above, appropriate education and training about AI algorithms not only positively influence beneficence but also empower autonomy, justice, and non-maleficence by enabling more informed decision-making, consideration of inequalities and biases, and more effective integration of AI into medical education. The training on AI may encompass tutorials, supervised workshops, and offline or online materials that help users understand how and when to use each application. On the other hand, working together in close collaboration with all stakeholders, including students, educators, AI developers, and healthcare professionals, is essential for the most beneficial implementation of AI in medical education, accounting for special needs among different disciplines, for example, when exploring which AI application can enhance the learning experience for each specialty [29]. Finally, the regular assessment and optimization of AI applications in medical education are crucial to ensure that these technologies are employed in a way that maximizes benefits (and minimizes potential harms) [51]. By systematically monitoring and reevaluating the impact of AI applications on the medical curriculum, for example, by providing an easily accessible tool for evaluation, institutions can make data-driven decisions on how to improve or modify the use of AI to better serve the educational needs of students and educators.

## Conclusions

**Fig. 1:**
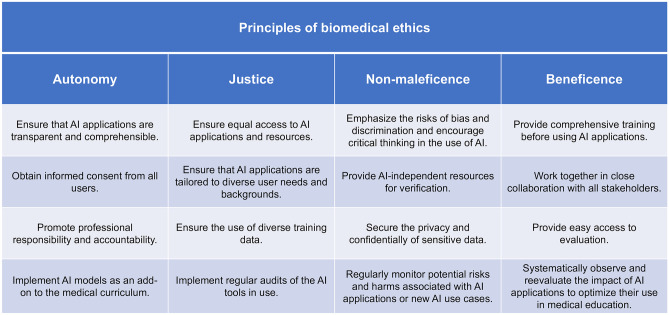
Overview of key recommendations for medical institutions towards integrating artificial intelligence (AI) into medical education, based on the four core principles of biomedical ethics — autonomy, justice, non-maleficence, and beneficence

A summary of all the recommendations developed within this comment can be viewed in Fig. 1. While integrating AI in medical education has the potential to provide a more immersive, interactive, and personalized learning experience, it is essential to establish a biomedical ethical framework beforehand, which should be regularly reevaluated and optimized based on user feedback and the latest developments in the field.

## Data Availability

Data sharing not applicable to this article as no datasets were generated or analyzed during the current study.
